# Supplementary material for: Neuronal gene expression in two generations of the marine parasitic worm, Cryptocotyle lingua
Source: Commun Biol. 2023 Dec 18;6:1279. doi: 10.1038/s42003-023-05675-4 (PMC10728431; doi:10.1038/s42003-023-05675-4)
Supplement: Supplementary file 2 — Supplementary Information [file 42003_2023_5675_MOESM2_ESM.pdf]

Supplementary information

Supplementary Note 1. **Supplementary behavior information.**

Here we illustrate the movement complexities, behavior state timing and speed of eight individual animals, offering insights into their activity patterns and behavioral nuances.

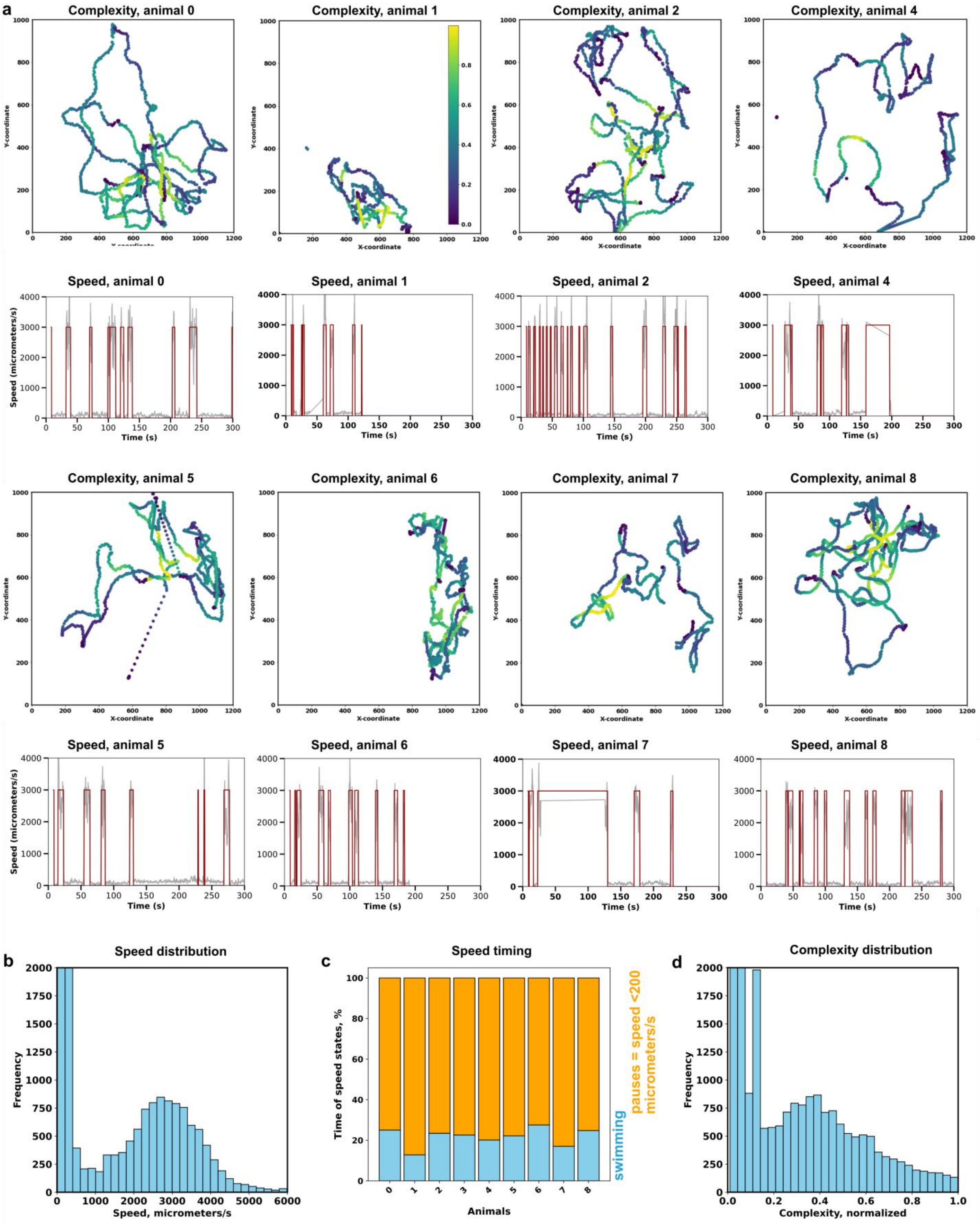

Supplementary Figure 1. **Visualization of behavior for each sampled freely moving cercaria larvae.** Note that animal 3 is plotted in the main figures (Fig. 1c).

(**a**) The trajectory complexity and the burst of speed of sampled cercariae. The trajectories are color-coded to represent the level of complexity (entropy) in the path of specific animals. Speed data are represented as binary signal (bursts of activity and quiescence) along with the speed line calculated from trajectories. (**b**) Speed distribution histogram for all sampled animals. (**c**) Timing of activity states for all sampled animals with the floating/quiescence swimming threshold at 200 micrometers/second. (**d**) Normalized complexity distribution histogram for the all sampled animals.

Supplementary Note 2. **Supplementary information on neuronal genes.**

Here we also characterize the domain structures and provide expression profiles for the identified neuronal proteins. Isolating parthenitae (redia) stage tissues independently is difficult due to the inseparable developing cercariae embryos within the maternal body. Consequently, our "redia" samples predominantly consist of cercarial tissues across all developmental stages. (Fig 1a-ii, Fig 4, 5). We hypothesize that some neuronal genes in mature larvae within rediae play roles in navigation and motility both within the snail host and during shedding.

Neurotransmitters biosynthesis and metabolism

We identified a group of cytoplasmic proteins homologues of enzymes involved in the biosynthesis and metabolism of classical neurotransmitters and small gaseous messengers in the transcriptome of *C. lingua* (Supplementary Data 2). Biopterin-dependent aromatic amino acid hydroxylases contain Biopterin_H catalytic domain that catalyzes the ring hydroxylation of aromatic amino acids and act as rate-limiting catalysts for catecholamines biosynthesis. We identified orthologs of biopterin-dependent aromatic amino acid hydroxylases, Tyrosine 3-monooxygenase and Tryptophan 5-hydroxylase 1 (*Cl*TH, *Cl*TPH1). Pyridoxal-dependent decarboxylase contain the conserved domain of group II pyridoxal-dependent decarboxylases and catalyses the decarboxylation of tryptophan to tryptamine, tyrosine into tyramine and histidine to histamine. We classified Aromatic-L-amino-acid decarboxylase and Tyrosine decarboxylase (*Cl*DDC, *Cl*TDC). The copper type II ascorbate-dependent monooxygenase family proteins contain Cu2 monooxygen domains that require

copper as a cofactor and which uses ascorbate as an electron donor for monooxygenase activity. We identified Dopamine beta-hydroxylase (*Cl*DBH) in *C. lingua* transcriptome. Aldehyde dehydrogenases contain aldehyde dehydrogenase domain and oxidize aliphatic and aromatic aldehydes including norepinephrine aldehyde using NADP as a cofactor. We classified Aldehyde dehydrogenase family 3 member A2 (*Cl*ALDH3A2). The choline/carnitine acyltransferase domain is found in a number of eukaryotic acetyltransferases including choline o-acetyltransferase an enzyme that catalyses the biosynthesis of the neurotransmitter acetylcholine. We identified ortholog of Choline acetyltransferase choline O-acetyltransferase (*Cl*CHAT) in the transcriptome of *C. lingua*. Carboxylesterase family type B proteins containing the carboxylesterase domain and act on carboxylic esters including acetylcholine. We classified orthologs of type-B carboxylesterase/lipase family Acetylcholinesterase and Cholinesterase 1,2 (*Cl*ACHE, *Cl*BCHE1, *Cl*BCHE2) involved in the in the transcriptome of *C. lingua*. Glutaminases contain eponymous glutaminase domain and deaminate glutamine to glutamate. We classified Glutaminase *Cl*GLS. Cys/Met metabolism PLP (pyridoxal-5'-phosphate)-dependent enzyme family contain eponymous domain and includes enzymes involved in cysteine and methionine metabolism which use PLP as a cofactor. We classified Cystathionine gamma-lyase *Cl*CTH. PLP-dependent enzymes superfamily contain a PLP domain and includes cysteine synthase. We identified Cystathionine beta-synthase *Cl*CBS in transcriptome of *C. lingua* (Supplementary Fig. 2b).

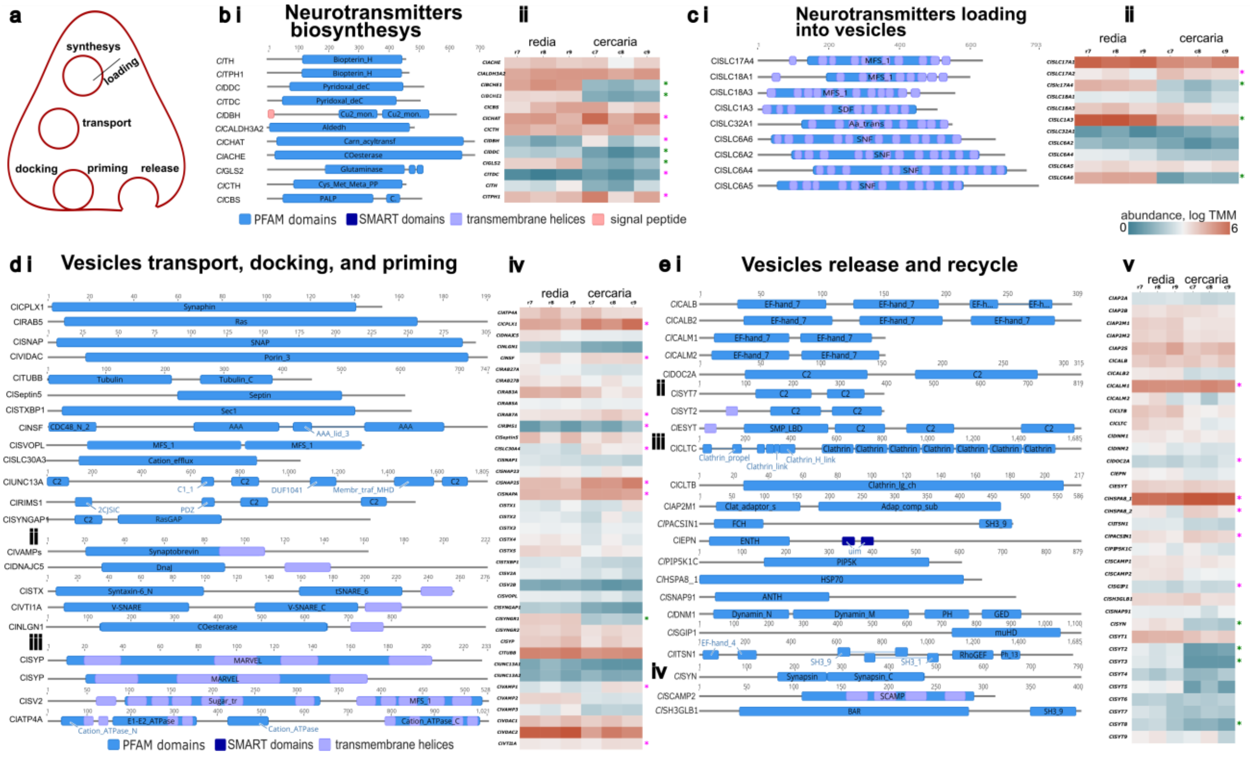

Supplementary Figure 2. **Characterized vesicle cycle proteins.** (**a)** Schematics of vesicle cycle. (**b**) Characterization of *C. lingua* proteins related to neurotransmitters biosynthesis and metabolism gene subset. Domain architecture of proteins (**i**) and the heat map of the transcript abundance levels (**ii**) in rediae and cercariae. Scale in a – protein length in amino acids (aa). Here and below transcript abundance levels in rediae and cercariae are shown as log of cross-sample normalized TMM. (**c**) Characterization of *C. lingua* proteins related to the subset of packaging and storage of neurotransmitters genes. Domain architecture of the proteins (**i**) and transcript abundance levels in rediae and cercariae (**ii**). **(d)** Characterization of *C. lingua* proteins related to transport , docking and priming of synaptic vesicles gene subset. Domain architecture of cytoplasmic proteins (**i**), single-pass membrane proteins (**ii**) and multi-pass membrane proteins (**iii**). Transcript abundance levels in rediae and cercariae (**iv**). **(e)** Characterization of *C. lingua* proteins related to release and recycle gene subset. Domain architecture of EF-Hand superfamily proteins **(i)**, double C2-like domain-containing proteins (**ii**), proteins involved in Clathrin-mediated endocytosis (**iii**) and proteins associated with small synaptic vesicles (**iv**); scale – protein length (aa). Transcript abundance levels of release and recycle gene subset (**v**) in rediae and cercariae. Differential expression: FDR <= 0.001 and FC>[1]; green apteryx – genes upregulated in redia, magenta apteryx – genes upregulated in cercaria.

Packaging and storage of neurotransmitters (Synaptic vesicles loading)

This group of genes encode multi-pass membrane proteins (Supplementary Data 3) bearing four characteristic PFAM domains. Major Facilitator Superfamily (MFS), dicarboxylate symporter family (SDF), Sodium:neurotransmitter symporter family (SNF) and Amino acid transporter (Aa_trans). Glutamate/aspartate transporters in the *C. lingua* transcriptome are represented by five proteins. These are three MSF-proteins, one homolog of Vesicular glutamate transporter (*Cl*Slc17A4) and two Sialin homologs (*Cl*SLC17A1, 2). SDF-bearing Excitatory amino acid transporter (*Cl*SLC1A3) and SNF-bearing Sodium- and chloride-dependent taurine transporter (*Cl*SLC6A6) were also classified within the group.

Monoamine transporters are represented by the MSF-bearing protein *Cl*SLC18A1, homologous to Synaptic vesicular amine transporter, and two SNF-bearing proteins: the *Cl*SLC6A2 which is similar to both the Sodium-dependent dopamine transporter and the Sodium-dependent norepinephrine transporter, and the *Cl*SLC6A4 homologous to Sodium-dependent serotonin transporter. We classified SNF-bearing *Cl*SLC6A5 and Aa_trans-bearing *Cl*SLC32A1 (homolog of Vesicular inhibitory amino acid transporter) as Glycine transporters. An MSF-protein *Cl*SLC18A3, the Vesicular acetylcholine transporter, was also identified in the *C. lingua* transcriptome (Supplementary Fig. 2c).

Transport, docking and priming of synaptic vesicles (Vesicle trafficking)

The first subset, cytoplasmic proteins, includes homologs of complexin (*Cl*CPLX1), alpha-soluble NSF attachment protein (*Cl*SNAP), synaptosomal-associated proteins Munc18 (*Cl*SNAP1), SNAP23, SNAP25 (*Cl*SNAP23,25), septin 5 (*Cl*septin5), syntaxin-binding protein (*Cl*STXBP1), NSF (N-ethylmaleimide sensitive) protein (*Cl*NSF), tubulin-beta (*Cl*TUBB), RAS oncogene family Rab3A, Rab5A, Rab7A, Rab27A,B members (*Cl*RAB3A, *Cl*RAB5A, *Cl*RAB7A, *Cl*RAB27A, *Cl*RAB27B), Regulating synaptic membrane exocytosis protein 1 (*Cl*RIMS1), Ras/Rap GTPase-activating protein SynGAP (*Cl*SYNGAP1), two homologous genes of voltage-dependent anion-selective channel (*Cl*VDAC1, 2), two Munc13a homologs (*Cl*UNC13A1, 2), Putative transporter SVOPL (*Cl*SVOPL) and synaptic vesicle associated Zinc transporter (*Cl*SLC30A4). The second, single-pass membrane proteins, consists of three homologs of vesicle-associated membrane protein (*Cl*VAMP1-3), homolog of cysteine string protein (*Cl*DNAJC5), vesicle transport through interaction with t-SNAREs homolog (*Cl*VTI1A), five genes encoding syntaxins (*Cl*STX1-5) and Neuroligin-1 homolog (*Cl*NLGN1). The third subset, multi-pass membrane proteins, includes synaptophysin (*Cl*SYP), two synaptogyrins (*Cl*SYNGR1, 2), sodium/potassium-transporting ATPase subunit alpha (*Cl*ATP4A) and two chains of synaptic vesicle glycoprotein 2 (*Cl*SV2A, B) (Supplementary Data 4, Supplementary Fig. 2d).

Vesicles release and recycle

We classified four EF-Hand superfamily proteins found in the transcriptome of *C. lingua* as Calbindin (*Cl*CALB), Calretinin/Calbindin2 (*Cl*CALB2) and two variants of Calmodulin (*Cl*CALM1, 2). The family of Double C2-like domain-containing proteins is represented by the ortholog of DOC2A (*Cl*DOC2A) and 10 homologs of Synaptotagmin (*Cl*SYT1-9, *Cl*ESYT). Among synaptotagmins, four proteins are cytoplasmic (*Cl*SYT6-9) and other six are membrane anchored including one homolog of Extended synaptotagmin (*Cl*ESYT). Proteins of Clathrin-associated complex involved in Clathrin-mediated endocytosis are rather diverse in domain architecture. They are represented with Clathrin heavy (*Cl*CLTC) and light (*Cl*CLTB) chains, Clathrin coat assembly protein (*Cl*SNAP91), Epsin (*Cl*EPN), two Dynamin homologs (*Cl*DNM1,2), Intersectin-1 (*Cl*ITSN1), Phosphatidylinositol 4-phosphate 5-kinase type-1 gamma (*Cl*PIP5K1C), SH3-containing GRB2-like

protein 3-interacting protein 1 (*Cl*SGIP1), AP-2 complex subunits (*Cl*AP2A1, B1, S1,M1,M2), two homologs of co-chaperone Heat shock cognate 71 kDa protein (ClHSPA8_1, 2) and Protein kinase C/casein kinase substrate neurons (*Cl*PACSIN1). In Platyhelminthes the homolog of the gene PACSIN1 is annotated as Antigen EG13. Finally, proteins associated with small synaptic vesicles, Synapsin (*Cl*SYN), two homologs of Secretory carrier-associated membrane protein 1 (*Cl*SCAMP1, 2) and SH3-domain GRB2-like endophilin B1 (*Cl*SH3GLB1) (Supplementary Data 5, Supplementary Fig. 2e).

Reception machinery

Neurotransmitter ligand-gated ion channels are transmembrane receptor-ion channel complexes and contain receptor family ligand binding region and neurotransmitter-gated ion-channel transmembrane region. These channels open transiently upon binding of specific ligands, allowing rapid transmission of signals at chemical synapses. We classified nicotinic acetylcholine receptors *Cl*ACC-1.1, *Cl*ACC-1.2, *Cl*ACC-2, *Cl*ACC-2.1, *Cl*ACC-2.2, *Cl*CHRACN1, *Cl*CHRACN2, *Cl*CHRACN3, *Cl*CHRACN4 and *Cl*CHRACN5 orthologs of the Acetylcholine gated chloride channels of *Schistosoma mansoni* ACC-1 and ACC-2 in the transcriptome studied. We also identified another inhibitory chloride ion channel Glycine receptors, both alpha and beta subunits *Cl*GLRA, *Cl*GLRA2, *Cl*GLRB. From this group of receptors we also classified Glutamate receptors, containing ligated ion channel L-glutamate-binding site excitatory cation channels kainite and N-methyl-D-aspartate (NMDA) - *Cl*GRIK1, *Cl*GRIK2, *Cl*GRIK3, *Cl*GRIK4, *Cl*GRID2, *Cl*GRIN1 and chloride channel *Cl*GLUCL.

Metabotropic receptors contain ligand binding ANF-receptor and seven transmembrane domains. We identified several putative g-protein coupled receptor families including orthologs of muscarinic acetylcholine receptors *Cl*GAR1 and *Cl*GAR2, orthologs of dopamine receptors *Cl*D2R1, *Cl*D2R2, orthologs of serotonin receptors *Cl*HTR1A1-A5, octopamine-tyramine receptors *Cl*Octbeta1 and *Cl*Oct2, and ortholog of Metabotropic glutamate receptor (GRM2-5) *Cl*GRM1. We also identified ortholog of Inositol 1,4,5-trisphosphate receptor type 1 *Cl*ITPR1,2 containing ion transport domain.

A portion of reception machinery in *C. lingua* transcriptome is represented by cytoplasmic proteins. The *Cl*SHANK1 contain Ferm Fo, ankyrin and PDZ domains and represents an ortholog of

an adapter protein SHANK1 that interconnects metabotropic glutamate receptors and the actin-based cytoskeleton. The *Cl*DLG1 is an ortholog of the Disks large homolog (DLG1-4) playing role in synaptogenesis and chemical synaptic transmission and contain a receptor targeting L 27, PDZ and guanylate kinase domains. The *Cl*HOMER1 is a ortholog of Homer protein (HOMER1,3) -- postsynaptic density scaffolding protein regulating synaptic metabotropic glutamate function and contain WASP homology region 1. *Cl*DBNL is an ortholog of Drebrin-like protein (DBN1) playing a role in neuron morphogenesis and synapse formation. It contains actin depolymerisation factor/cofilin -like and Src homology 3 domains. The *Cl*STRN1 is an ortholog of Striatin (STRN), a calmodulin-binding protein which may function as scaffolding or signaling protein and may play a role in dendritic Ca2+ signaling and contain eponymous domain. We also identified the *Cl*DLGAP1 containing an eponymous domain an ortholog of Disks large-associated protein (DLGAP1-4) which is a part of the postsynaptic scaffold in neuronal cells. We also classified orthologs of P2X purinoreceptor *Cl*P2X1, *Cl*P2X2 containing eponymous domain and of Guanylate cyclase soluble subunit beta-1 containing NO binding HNOB domain (Supplementary Data 6, Supplementary Fig. 3a).

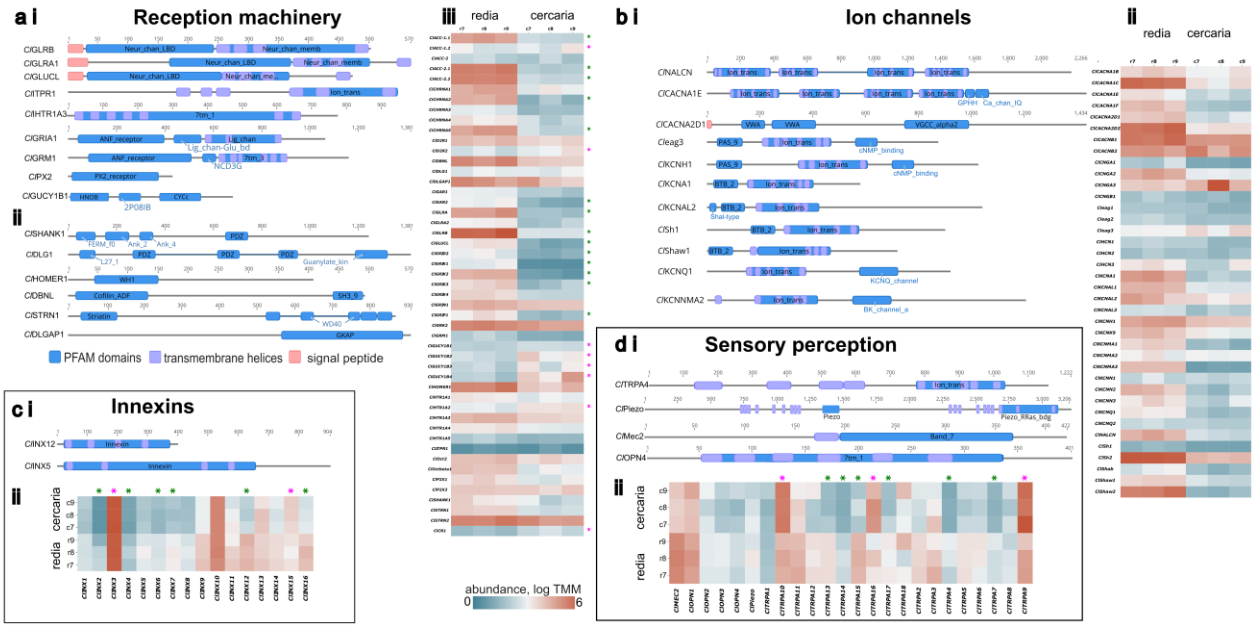

Supplementary Figure 3. **Characterized receptor, electric properties machinery, and sensory proteins.** (**a**) Characterization of *C. lingua* proteins related to reception machinery gene subset. Domain architecture of representative multi-pass membrane proteins (**i**) and cytoplasmic proteins (**ii**); scale – protein length amino acids (aa). Transcript abundance levels in rediae and cercariae (**iii**). (**b**). Characterization of *C. lingua* ion channels gen subset. Domain architecture of sodium leak channel, potassium and calcium voltage gated channels (**i**) and their expression abundance in rediae and cercariae (**ii**). (**c**) Characterization of *C. lingua* innexins. Domain architecture of innexins (**i**) and their expression profiles (**ii**). (**d**) Characterization of *C. lingua* sensory gene subset. Domain architecture of proteins (**i**) and

their transcript abundance in rediae and cercariae (**ii**). Scale – protein length (aa). Differential expression: FDR <= 0.001 and FC>[1]; green apteryx – genes upregulated in redia, magenta apteryx – genes upregulated in cercaria.

Ion channels

Of all Na^+ channel proteins only a sodium leak channel non-selective protein (*Cl*NALCN) was identified. Domain architectures of *C. lingua* voltage-dependent calcium channels, R-type subunit alpha-1E (*Cl*CACNA1E) N-type subunit alpha-1B (*Cl*CACNA1B), L-type subunit alpha-1C (*Cl*CACNA1C) and L-type subunit alpha-1F (*Cl*CACNA1F) are similar, hallmarked with C-terminal domains GPHH, Ca_chan_IQ (Supplementary Fig. 3b) (*Cl*CACNA1E). The alpha-2/delta subunits of voltage-dependent calcium channels (*Cl*CACNA2D1 and *Cl*CACNA2D2) are characterized with two N-terminal von Willebrand factor (vWF) type A domains and a specific C-terminal domain VGCC_alpha2.

We identified some of *C. lingua* potassium voltage-gated channels , encoding three orthologs of protein eag (Cleag1 - 3), subfamily H member (*Cl*KCNH1), subfamily A member (*Cl*KCNA1), three proteins Shal (*Cl*KCNAL1 - 3) with characteristic N-terminal domain Shal-type, two proteins Shaker (*Cl*Sh1, 2) homologues to functionally characterized SKv1.1 channel, protein Shab (*Cl*Shab), two proteins Shaw (*Cl*Shaw1, 2), two members of KQT-subfamily (*Cl*KCNQ1, 2), four transmembrane inward-rectifying TWIK-related potassium channel (*Cl*KCNK9) and three potassium/sodium hyperpolarization-activated cyclic nucleotide-gated channels (*Cl*HCN1-3). Among three types of Ca2+-activated K+ channel we classified homologs of small-conductance (SK) type characterized by the SK channel and Ca2+-binding protein calmodulin (CaM) domain (*Cl*KCNN1-3) and large conductance type with BK channel a domain (*Cl*KCNMA1-3). We also classified homologs of cGMP-gated cation channel subunits (*Cl*CNGA1-3, *Cl*CNGB) (Supplementary Data 8, Supplementary Fig. 3b).

Innexins

Innexins forming gap junctions and non-junctional membrane channels were found expressed in *C. lingua.* They bear a hallmark domain with four transmembrane helices. Innexins were represented

in the transcriptome by 16 genes (*Cl*INX1 - 16). Only two of them were expressed at levels above 100 TMMs (Supplementary Data 7, Supplementary Fig. 3c).

Sensory perception proteins

We identified orthologs of transient receptor ion channels *Cl*TRPA1-A18 containing transient receptor ion channel, polycystin cation channel, ion transport protein domains. We classified ortholog of another non-specific cation channel, Piezo mechanosensitive ion channel (*Cl*Piezo) containing eponymous domains and ortholog of Mechanosensory protein 2 mec-2 containing prohibitin homologues domain *Cl*MEC2 . We also identified orthologs of light-absorbing opsins *Cl*OPN1-4 from the ion transport protein members of the seven-transmembrane-domain proteins of the G protein-coupled receptor (GPCR) superfamily (Supplementary Data 8, Supplementary Fig. 3d).
