# Supplementary material for: Neuronal gene expression in two generations of the marine parasitic worm, Cryptocotyle lingua
Source: Commun Biol. 2023 Dec 18;6:1279. doi: 10.1038/s42003-023-05675-4 (PMC10728431; doi:10.1038/s42003-023-05675-4)
Supplement: Supplementary file 3 — Description of Additional Supplementary Files [file 42003_2023_5675_MOESM3_ESM.pdf]

Description of Additional Supplementary Files

File name: Supplementary Data 1-9
Description: List of Homeobox and neuronal genes with reference proteins similarity info and expression profiles. Data is organized in tabs per groups of described genes.

File name: Supplementary Data 10
Description: Sequences of HCR in situ probes used.

File name: Supplementary Data 11
Description: The source data behind the graphs in the paper.

File name: Supplementary Movie 1
Description: Spontaneous movements of C. lingua rediae.

File name: Supplementary Movie 2
Description: Spontaneous swimming of C. lingua cercaria.
